# Improving Linkage to Care of Hepatitis C: Clinical Validation of GeneXpert^®^ HCV Viral Load Point-of-Care Assay in Indonesia

**DOI:** 10.4269/ajtmh.20-1588

**Published:** 2021-05-17

**Authors:** Meta Dewi Thedja, Dhita Prabasari Wibowo, Korri Elvanita El-Khobar, Susan Irawati Ie, Turyadi, Lyana Setiawan, Ignatia Sinta Murti, David Handojo Muljono

**Affiliations:** 1 Eijkman Institute for Molecular Biology, Jakarta, Indonesia;; 2 Faculty of Medicine, Universitas Hasanuddin, Makassar, Indonesia;; 3 Virology Laboratory, Dharmais National Cancer Hospital, Jakarta, Indonesia;; 4 Division of Gastroenterology, Department of Internal Medicine, Abdoel Wahab Sjahranie Regional General Hospital, Samarinda, Indonesia;; 5 Faculty of Medicine and Health, University of Sydney, New South Wales, Australia

## Abstract

Hepatitis C virus (HCV) infection large-scale diagnosis and treatment are hampered by lack of a simple, rapid, and reliable point-of-care (POC) test, which poses a challenge for the elimination of hepatitis C as a public health problem. This study aimed to evaluate Cepheid Xpert^®^ HCV Viral Load performance in comparison with the Roche Cobas^®^ TaqMan^®^ HCV Test using serum samples of HCV-infected patients in Indonesia. Viral load quantification was performed on 243 anti-HCV positive patients’ samples using both Xpert HCV VL and Roche HCV tests, followed by HCV genotyping by reverse hybridization. Strength of the relationship between the assays was measured by Pearson correlation coefficient, while level of agreement was analyzed by Deming regression and Bland–Altman plot analysis using log_10_-transformed viral load values. Quantifiable viral load was detected in 180/243 (74.1%), with Xpert HCV VL sensitivity of 100% (95% CI 0.98, 1.00) and specificity of 98.4% (95% CI 0.91, 0.99) based on the Roche HCV test, while HCV genotypes were determined in 172/180 (95.6%) samples. There was a good correlation between both assays (*r* = 0.97, *P* < 0.001), overall and per genotype, with good concordance by Deming regression and a mean difference of −0.25 log_10_ IU/mL (95% CI −0.33, −0.18) by Bland–Altman plot analysis. Xpert HCV VL test was demonstrated as a POC platform with good performance for HCV diagnosis and treatment decision that would be beneficial for decentralized services in resource-limited areas. HCV testing sites, alongside additional GeneXpert modular systems distributed toward the fight against COVID-19, could ensure some continuity, once this pandemic is controlled.

## INTRODUCTION

Globally, an estimated 170 million people have serological evidence of current or past hepatitis C virus (HCV) infection, and 71 million people have chronic viremic infection.^1^ Approximately 399,000 people die each year from hepatitis C, mostly from cirrhosis and hepatocellular carcinoma (HCC).^1,2^ This disease imposes a great multifaceted economic burden worldwide that includes direct medical expenses and indirect costs because of impaired quality of life and loss of work productivity.^3^ In response to this concern, the World Health Organization (WHO) developed the Global Health Sector Strategy (GHSS) on Viral Hepatitis 2016–2021. This strategy outlines a set of service coverage targets: diagnosing 90% of chronic infections, treating 80% of eligible people, accomplishing a global impact, and achieving a 90% reduction in new chronic infections and a 65% reduction in mortality by 2030. The aim is the elimination of viral hepatitis as a major public health threat.^4^

The advent of direct-acting antiviral (DAA) drugs for HCV heralded a significant breakthrough for hepatitis C treatment, providing an opportunity to achieve the targeted global HCV elimination.^5^ The U.S. Food and Drug Administration (FDA) and the European Medicines Agency (EMA) had approved 13 DAAs and several fixed-dose combinations for the treatment of HCV infection. The approval of pangenotypic regimens (sofosbuvir/velpatasvir, sofosbuvir/velpatasvir/voxilaprevir, and glecaprevir/pibrentasvir) has reduced the need for genotyping to guide treatment decisions. These regimens have shown high efficacy across all six major HCV genotypes and the newly discovered genotypes 7 and 8.^6,7^

Despite the increased options, expansion of access, and steep price reduction of DAAs, only 20% of infected persons have been diagnosed and 7% have received treatment worldwide, with the majority in higher income settings.^8^ In many low- and middle-income countries (LMICs), less than 1% of infected people have been diagnosed and treated. While the world attention is focused on the final steps in the cascade of care for HCV infection, uptake of DAA treatment is progressing slowly and unevenly.^9,10^ Complex clinical management and the cost of HCV testing appear as major impediments to the screening and diagnostic coverage.^6^ These conditions, along with the need to test samples in batches with long turn-around time and the potential for loss to follow-up, may impede the wide screening and diagnosis of HCV infection. To support the scale-up of the HCV elimination program, there is an urgent need for alternative choices involving simple, rapid, and reliable point-of-care (POC) viral load tests that can be better suited for decentralized services, linking diagnosis to care.^11–13^

Several POC assays (including venipuncture-based testing, finger-stick capillary whole-blood testing, and oral fluid diagnostic testing) that facilitate HCV RNA confirmation in a single visit are currently available or in the last-stage development.^14,15^ Of these assays, Xpert^®^ HCV Viral Load (Cepheid, Sunnyvale, CA) has been CE-marked and WHO-prequalified for use in resource-limited settings.^16–18^ Nucleic acid extraction, amplification, and detection of target sequences are carried out in a cartridge and processed in a GeneXpert^®^ Instrument, producing a quantitative HCV RNA result in 105 minutes. This Cepheid system is semiportable, implementable with minimal laboratory set-up, and does not require batch testing. It is also a modular platform that enables testing for other infections.^19^ In search of POC diagnostic tools suitable for decentralized service of hepatitis C, we evaluated the performance of the Xpert^®^ HCV viral load test and compared it with the FDA-approved Roche Cobas® TaqMan^®^ HCV v2.0 test,^20^ as the widely used HCV testing platform in Indonesia, using Indonesian HCV-samples with various genotypes.

## MATERIALS AND METHODS

### Study participants and clinical samples.

This was a prospective study conducted at the Hepatitis Research Unit, Eijkman Institute for Molecular Biology, Jakarta, Indonesia. Between June 1, 2018 and January 31, 2019, 243 anti-HCV positive patients who were either referred to the Eijkman Institute or coming to the outpatient department of Dharmais Cancer Hospital in Jakarta—a national referral center for cancer diagnosis and treatment—or Abdoel Wahab Sjahranie Regional General Hospital in Samarinda, Kalimantan Island, were consecutively enrolled. Data were collected on patient age, gender, previous HCV treatment history, as well as results of transient elastography (TE) using FibroScan^®^, which defines cirrhosis as having a value of 14.1 kPa or higher (fibrosis stage 4).^21^

After written consent was obtained, venous whole blood was collected from each eligible patient in a 9-mL ethylene-diamine-tetraacetic acid (EDTA) tube. After centrifugation, plasma was extracted from each tube and divided into four 1.2-mL aliquots—two were kept for viral load testing, one for genotype determination, and one for backup. The study was in accordance with and approved by the Eijkman Institute Research Ethics Commission (EIREC No. 115/2017). Written informed consent was obtained from each patient.

### Xpert® HCV viral load testing.

The Xpert^®^ HCV viral load test (hereafter referred to as Xpert HCV VL) was performed by laboratory technicians who were recruited from district laboratories and were directly supervised by trained research scientists at the Eijkman Institute as part of capacity-building for decentralization of HCV care in the country. Briefly, a total of 1000 μL plasma was placed into the Xpert cartridge, which was scanned and loaded into the GeneXpert® instrument according to the manufacturer’s instructions. Results were recorded as undetected when viral load was below 4 IU/mL (0.6 log_10_ IU/mL), detected below 10 IU/mL (1.0 log_10_ IU/mL), or above 10^8^ IU/mL (8.0 log_10_ IU/mL)—which is the lower and upper limits of quantification (LOQ), or within the LOQ (between 1.0 log_10_ IU/mL and 8.0 log_10_ IU/mL), defined as quantifiable.^22^

### Cobas^®^ TaqMan^®^ (Roche) HCV RNA viral load testing and HCV genotype determination.

A plasma sample (850 μL) was tested for viral load using the COBAS^®^ AmpliPrep/Cobas^®^ TaqMan^®^ HCV Quantitative Test v2.0 (hereafter referred to as Roche HCV) according to the manufacturer’s instruction on Cobas^®^ Taqman^®^ 48 instrument. Results were recorded as undetected, detected under 15 IU/mL (1.2 log_10_ IU/mL), or above 10^8^ IU/mL (8.0 log_10_ IU/mL) outside the range of the LOQ, or detected within the LOQ (between 1.2 log_10_ IU/mL and 8.0 log_10_ IU/mL), defined as quantifiable.^22^ One aliquot of all samples with detectable HCV RNA by the Roche HCV platform was tested for HCV genotype (GT) by a second-generation LiPA-HCV genotype assay (Versant HCV Genotype 2.0; Siemens Healthcare Diagnostics).^23^

### Statistical analysis.

Baseline characteristics of the patients were summarized descriptively. The sensitivity and specificity of the Xpert HCV VL was assessed qualitatively using both detectable and quantifiable thresholds (LOQ > 1.0 log_10_ IU/mL) compared with the Roche assay as the reference (LOQ > 1.2 log_10_ IU/mL), because the Roche assay is currently the widely used HCV testing platform in Indonesia. Undetectable or detectable viral load under the lower LOQ (hereafter referred to as unquantifiable) of respective platforms was considered as a negative result, whereas viral load within or over the LOQ boundaries was considered as a positive result. The strength of the relationship between the two assays was measured by the Pearson correlation coefficient, while the level of agreement was analyzed by Deming regression and the Bland–Altman plot analysis using the log_10_-transformed viral load values in IU/mL. Deming regression takes account of measurement errors for both methods,^24^ while the Bland–Altman plot measures the mean difference (bias) and the concordance, including limits of agreement (LOA) and their 95% confidence intervals (CI) between the quantification results of both assays.^25^

Samples with unquantifiable viral load on either platform were excluded from the quantitative analysis. The performance of the Xpert HCV VL test for different genotypes was also analyzed. Tests were two sided, and *P* values < 0.05 were considered statistically significant. Data were analyzed using the Statistical Program for Social Sciences (IBM SPSS version 22.0 for Windows; SPSS, Chicago, IL) and NCSS Statistical Software version 12 (NCSS, Kaysville, UT).

## RESULTS

### Characteristics of study population.

Among all enrolled participants (*n* = 243), the median age was 49 years, 64.6% (*n* = 157) were male, and 48 (19.8%) had previous history of HCV treatment. Fibrosis assessment by transient elastography (TE) was performed on 66 patients; of these, 20 (30.3%) had cirrhosis. Among 180 patients with quantifiable viral load on Roche HCV, HCV genotypes were successfully determined on 172 samples, with GT1 (108/62.8%) and GT3 (26/15.1%) being the most common, followed by GT2 (20/11.6%), GT4 (17/9.9%), and GT6 in one (0.6%) sample (Table 1).

**Table 1:** Characteristics of study population (*n* = 243)

Characteristics	n (max, min)	%
Age (years)*	49 (10, 87)	
Male	157	64.6
Fibrosis stage† (*n* = 66)		
F0 (< 5.1 kPa)	3	4.5
F1 (≥ 5.1 and < 7.2 kPa)	4	6.1
F2 (≥ 7.2 and < 9.6 kPa)	19	28.8
F3 (≥ 9.6 and < 14.1 kPa)	20	30.3
F4 (≥ 14.1 kPa)	20	30.3
History of HCV treatment (*n* = 48)‡		
IFN or PegIFN	44	91.7
DAA§	7	14.6
Both IFN/PegIFN and DAA	3	6.3
HCV genotypes (*n* = 172)‖		
**1**	108	62.8
1a	57	33.1
1b	40	23.3
Subtype unknown	11	6.4
**2**	20	11.6
2a/c	14	8.1
Subtype unknown	6	3.5
**3**	26	15.1
3a	15	8.7
3k	11	6.4
**4**	17	9.9
4h	6	3.5
Subtype unknown	11	6.4
**6**	1	0.6
6c	1	0.6
Indeterminate	8	4.7

* Median (minimum, maximum).

† By transient elastography (TE) using FibroScan^®^, cirrhosis was defined as having a TE value of 14.1 kPa or above (fibrosis stage 4) [31].

‡ With previous HCV treatment.

§ Either daclatasvir/sofosbuvir or sofosbuvir/ledipasvir with or without ribavirin.

‖ Genotypes among 180 patients with quantifiable viral load by Roche HCV.

### Sensitivity and specificity of Xpert HCV VL.

Of the 243 patients, 180 (74.1%) had detectable and quantifiable viral load above the lower LOQ on both platforms, and 55 (22.6%) had undetectable viral load on both platforms (Table 2). One sample was unquantifiable and detectable by Roche HCV but quantifiable by Xpert HCV VL with a viral load of 1.04 log_10_ IU/mL. This was one of the eight samples with indeterminate HCV genotype. There were no samples quantifiable by Roche HCV but unquantifiable by Xpert HCV VL. Considering quantifiable viral load as positive and unquantifiable viral load as negative results, the sensitivity of Xpert HCV VL was 100% (95% CI 0.98, 1.00) and the specificity was 98.4% (95% CI 0.91, 0.99) compared with the Roche HCV test. Viral load values and bias between the two tests at percentiles are shown in Table 3.

**Table 2:** Comparison of viral load test results between Xpert HCV VL and Roche HCV VL (*n* = 243)

		Roche Cobas^®^ TaqMan^®^ HCV test			
		Detectable and quantifiable	Detectable and unquantifiable	Undetectable	Total
Xpert^®^ HCV VL test	Detectable and quantifiable	180	1	0	181
	Detectable and unquantifiable	0	7	0	7
	Undetectable	0	0	55	55
	Total	180	8	55	243

**Table 3:** Viral load values and bias between Xpert HCV VL and Roche HCV tests at percentiles

Variable	Xpert HCV viral load	Roche HCV	Bias
	(95% CI)	(95% CI)	(95% CI)
	(log_10_ IU/mL)	(log_10_ IU/mL)	(log_10_ IU/mL)
10th percentile	4.56 (3.69, 4.83)	4.43 (4.01, 4.88)	−0.90 (−1.03, −0.79)
25th percentile	5.35 (5.14, 5.48)	5.42 (5.13, 5.76)	−0.55 (−0.68, −0.49)
50th percentile	5.95 (5.75, 6.09)	6.19 (5.98, 6.37)	−0.26 (−0.36, −0.20)
75th percentile	6.42 (6.34, 6.52)	6.76 (6.58, 6.86)	0.04 (−0.06, 0.18)
90th percentile	6.70 (6.62, 6.85)	7.24 (7.06, 7.44)	0.39 (0.25, 0.54)

CI = confidence interval.

### Concordance between Xpert HCV VL and Roche HCV.

**Figure 1.**
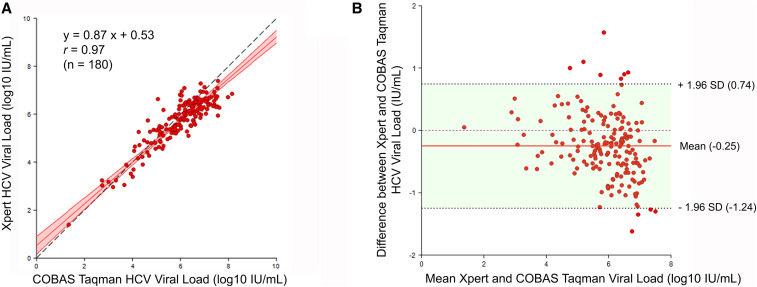
Deming regression and Bland-Altman plot analyses for samples’ HCV quantification by Xpert and Roche assays. (**A**) The Deming regression plot shows the Deming fitted regression line (red), associated confidence interval bounds (red shadow), and Pearson’s correlation. The black dashed line represents the 45° Y = X line (identity line). (**B**) The Bland–Altman plot shows the difference between the HCV RNA levels obtained by the two assays; the mean difference is depicted by the red line; and dotted lines indicate the upper and lower limit of agreements (LOAs) corresponding to ± 1.96 standard deviation. This figure appears in color at www.ajtmh.org.

**Figure 2.**
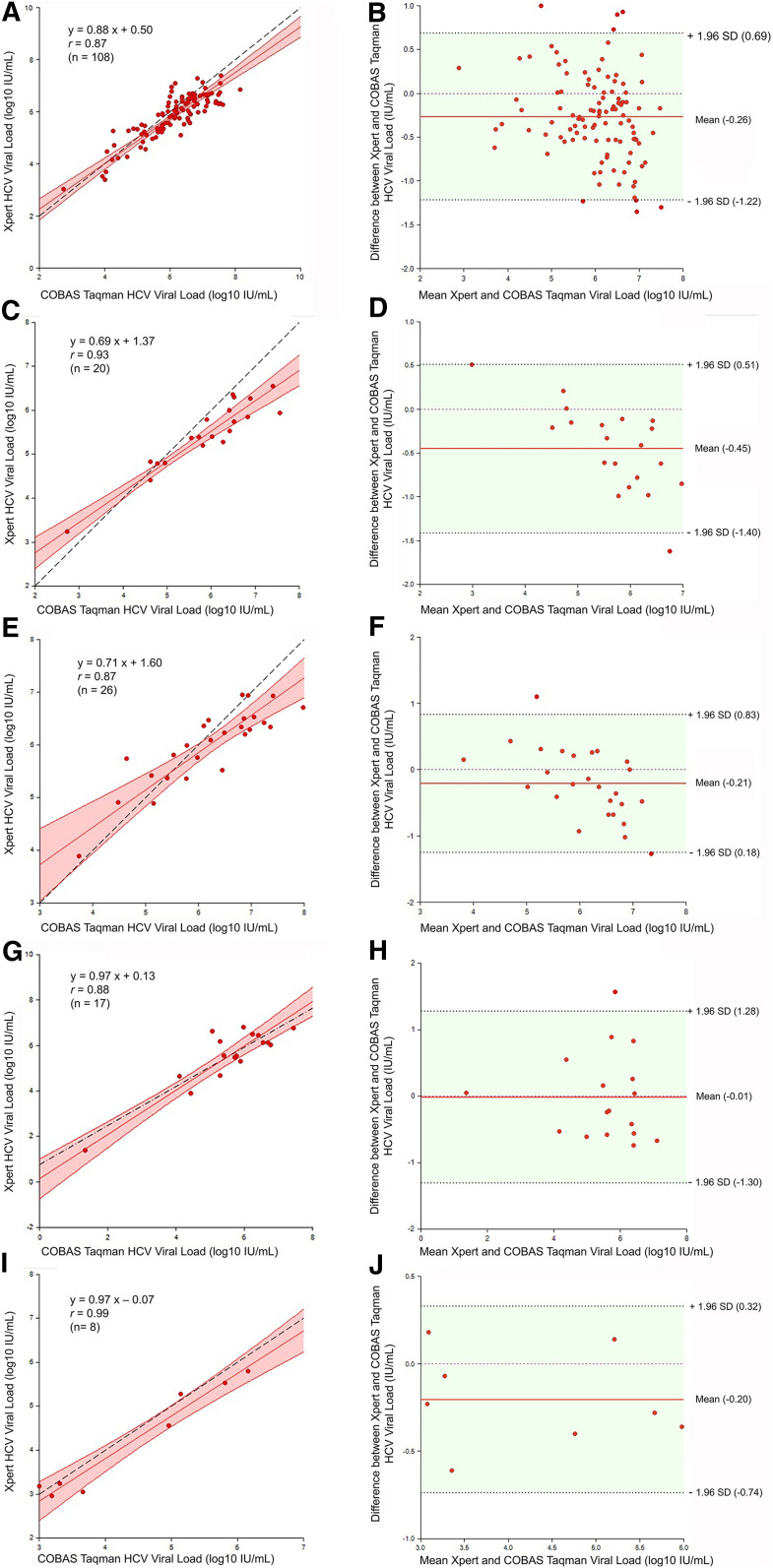
Deming regression and Bland-Altman plot analyses by HCV genotype. Deming regression plot and correlation of genotype 1 samples (**A**), genotype 2 samples (**C**), genotype 3 samples (**E**), genotype 4 samples (**G**), and indeterminate genotype samples (**I**). Bland-Altman plot of genotype 1 samples (**B**), genotype 2 samples (**D**), genotype 3 samples (**F**), genotype 4 samples (**H**), and indeterminate genotype samples (**J**). The Deming regression plots show the Deming fitted regression lines (red), associated confidence interval bounds (red shadow), Pearson’s correlation, and the identity line (45° Y = X) (black dashed). The Bland–Altman plots show the mean HCV viral load of the two platforms (Xpert and Roche) against the difference in viral load values (Xpert minus Roche); the central horizontal line (red) indicates the mean difference, and the dotted lines indicate the upper and lower limits of agreement (LOAs) corresponding to ± 1.96 standard deviation. This figure appears in color at www.ajtmh.org.

A good correlation was seen between Xpert HCV VL and Roche HCV tests (Pearson’s correlation coefficient (*r*) = 0.97, *P* < 0.001). When analyzed separately by HCV genotype, the correlation was significant in GT1 (*r* = 0.87, *P* < 0.001), GT2 (*r* = 0.93, *P* < 0.001), GT3 (*r* = 0.87; *P* < 0.001), GT4 (*r* = 0.88, *P* < 0.001), and indeterminate genotype (*r* = 0.99, *P* < 0.001). Further analysis by Deming regression for the 180 samples showed the overall Deming regression equation *Y* = 0.87*X* + 0.53 (Figure 1A). When analyzed by genotype, the Deming regression equations were *Y* = 0.88*X* + 0.50 for GT1, *Y* = 0.69*X* + 1.37 for GT2, *Y* = 0.71*X* + 1.60 for GT3, *Y* = 0.97*X* + 0.13 for GT4, and *Y* = 0.97*X* − 0.07 for indeterminate genotype (Figure 2A, 2C, 2E, 2G, 2I). Detailed correlation and Deming regression results are shown in Table 4.

**Table 4:** Pearson’s correlation and Deming regression analysis of Xpert HCV VL against Roche HCV tests

Genotype	N	Pearson’s correlation		Deming regression			
		r	*P* value	Intercept (95% CI)	Slope (95% CI)	T-value	Df
All	180	0.97	< 0.001	0.70 (0.32, 1.08)	0.84 (0.79, 0.90)	1.97	178
GT1	108	0.87	< 0.001	0.71 (0.11, 1.31)	0.84 (0.74, 0.94)	1.98	106
GT2	20	0.93	< 0.001	1.54 (0.84, 2.25)	0.66 (0.53, 0.80)	2.10	18
GT3	26	0.87	< 0.001	1.96 (0.59, 3.35)	0.65 (0.44, 0.86)	2.06	24
GT4	17	0.88	< 0.001	0.16 (−0.79, 1.11)	0.97 (0.79, 1.45)	2.13	15
GT6	1	NA	NA	NA	NA		
Indeterminate	8	0.99	< 0.001	−0.06 (−1.06, 0.93)	0.97 (0.77, 1.17)	2.45	6

CI = confidence interval; Df = N-2 degrees of freedom; NA = not applicable.

By the Bland–Altman plot analysis, the mean difference between the two platforms was −0.25 log_10_ IU/mL (95% CI −0.33, −0.18), with differences between the platforms ranging from −1.62 to 1.56 log_10_ IU/mL. The lower and upper LOAs were −1.24 log_10_ IU/mL (95% CI −1.37, −1.12) and 0.74 log_10_ IU/mL (95% CI 0.61, 0.87), respectively. One hundred sixty-nine (93.9%) samples were within the LOA, while eleven (6.1%) samples (five GT1, one GT2, two GT3, and three GT4) were outside the LOA (Figure 1B). By genotype, the mean differences across the two platforms were −0.26 ± 0.49 log_10_ IU/mL (95% CI −0.36, −0.17) for GT1, −0.45 ± 0.49 log_10_ IU/mL (95% CI −0.68, −0.22) for GT2, −0.21 ± 0.52 log_10_ IU/mL (95% CI −0.42, 0.00) for GT3, −0.01 ± 0.66 (95% CI −0.35, 0.33) log_10_ IU/mL for GT4, and −0.20 ± 0.27 log_10_ IU/mL (95% CI −0.43, 0.02) for indeterminate genotype. Detailed Bland–Altman analysis results are shown in Figure 2B, 2D, 2F, 2H, 2J, and Table 5.

**Table 5:** Bland-Altman Plot analysis of Xpert HCV VL against Roche HCV tests

Genotype	N	Mean difference ± SD (95% CI)	Lower LOA (95% CI)	Upper LOA (95% CI)	Inside LOA	Outside LOA
		(log_10_ IU/mL)	(log_10_ IU/mL)	(log_10_ IU/mL)	n (%)	n (%)
All	180	−0.25 ± 0.51 (−0.33, −0.18)	−1.24 (−1.37, −1.12)	0.74 (0.61, 0.87)	169 (93.9)	11 (6.1)
GT1	108	−0.26 ± 0.49 (−0.36, −0.17)	−1.22 (−1.37, −1.06)	0.69 (0.53, 0.84)	100 (92.6)	8 (7.4)
GT2	20	−0.45 ± 0.49 (−0.68, −0.22)	−1.40 (−1.81, 1.01)	0.51 (0.11, 0.91)	19 (95.0)	1 (5.0)
GT3	26	−0.21 ± 0.52 (−0.42, 0.00)	0.18 (−1.61, 0.88)	0.83 (0.45, 1.20)	24 (92.3)	2 (7.7)
GT4	17	−0.01 ± 0.66 (−0.35, 0.33)	−1.30 (−1.89, −0.71)	1.28 (0.67, 1.87)	16 (94.1)	1 (5.9)
GT6	1	NA	NA	NA	NA	NA
Indeterminate	8	−0.20 ± 0.27 (−0.43, 0.02)	−0.744 (−1.14, −0.33)	0.32 (−0.08, 0.74)	8 (100.0)	0 (0.0)

CI = confidence interval; LOA = limits of agreement; NA = not applicable.

## DISCUSSION

Spread across more than 17,000 islands, Indonesia has the highest number of HCV prevalence in Southeast Asia; an estimated 1,289,000 Indonesian people had a viremic infection in 2015.^26^ Beginning in 2017, a government-assisted program provided free testing and DAA treatment to 6,000 patients with active HCV infection in seven provinces in Indonesia. This program was gradually expanded to other provinces.^27^ Newly installed GeneXpert devices, together with those placed by a tuberculosis program, have been integrated to support scaling-up an HCV elimination program in Indonesia.^28^

This study showed Xpert HCV VL assay accurately quantified HCV viral load compared with the Roche HCV RNA assay, a leading assay used in Indonesia and worldwide.^29^ The sensitivity of the Xpert HCV VL for viral load measurement was found to be 100% (95% CI 97.9, 100.0). This finding confirms the studies of Iwamoto et al. among mostly GT1 and GT6 patients with a sensitivity of 100% (95% CI, 99.2, 100.0) in comparison to the Roche COBAS^®^ Ampliprep/Cobas^®^ TaqMan^®^ HCV Quantitative Test v2.0,^22^ those of Gupta et al. at 94.4% (95% CI 48.8, 99.8),^30^ and McHugh et al. at 98.0% (95% CI 96.1, 99,1),^31^ among mostly GT1 and GT3 patients against the Abbott RealTime HCV assay. No false negativity by Xpert HCV VL was seen in this study. We found a specificity of 98.4% (95% CI 0.91, 0.99) compared with 98.5% (95% CI 94.8, 99.8), 100% (95% CI 88.1, 100.0), and 98.1% (95% CI 95.2, 99.5) in the Iwamoto, Gupta, and McHugh’s studies, respectively.^22,30,31^ This assay provides a rapid, simple, and accurate POC molecular test for HCV viremia, fulfilling the requirements published by the Foundation for Innovative New Diagnostics FIND/WHO (diagnostic sensitivity > 95% and specificity > 98%).^18^ With minimal requirement of infrastructure and less turn-around time (105 minutes) than that of Roche HCV (around 4 hours), this test would be ideal for decentralization of molecular testing in a resource-limited setting.

Quantitative analysis revealed a significant correlation (*r* = 0.97, *P* < 0.001) between the Xpert HCV VL and Roche HCV, which was also comparable when calculated within individual genotypes. The Xpert HCV VL bias against the Roche HCV values varies from −0.90 log _10_ IU/mL at the 10th percentile to 0.39 log_10_ IU/mL at the 90th percentile. The negative bias at the lower end is likely because of sparse data in the range of the 10th to 25th percentiles, while data for higher viral load across the range of the 50th to 75th percentiles are more equally distributed, and therefore the regression curve is less driven by the high-end samples.

Bland–Altman analysis showed a mean difference of −0.25 ± 0.51 log_10_ IU/mL (95% CI −0.33, −0.18) between the two assays; the LOA was between −1.24 log_10_ IU/mL to 0.74 log_10_ IU/mL, with 11 (6.1%) of samples falling outside the LOA. Our overall LOA was wider compared with previous studies by Iwamoto (mean difference −0.01 log_10_ IU/mL; LOA −0.76 and 0.73),^22^ Gupta (mean difference 0.04 log_10_ IU/mL; LOA −0.42 and 0.49),^30^ McHugh (mean difference 0.03 log_10_ IU/mL; LOA −0.41 and 0.47),^31^ and Grebely (mean difference −0.036 log_10_ IU/mL; LOA −0.28 to 0.35).^32^ This could be attributable to smaller number of samples, particularly in the range of lower viral load values.^33,34^

Assays can perform differently by genotype,^35–37^ while detection of HCV RNA and measurement of viral load for the different genotypes are crucial to clinical management of HCV-infected patients.^38–40^ This study showed high efficiency and accuracy of the Xpert HCV VL assay for quantitation of HCV RNA GT1, GT2, GT3, GT4, and even indeterminate genotype.

The indeterminate HCV genotype samples found in our cohort may reflect the limitation of the LiPA test in accurately genotyping HCV genotype 6 samples—a prevalent genotype in Indonesia and Southeast Asian countries with many identified subgenotypes.^41,42^ Additional Sanger sequencing after a LiPA genotyping test might be beneficial in resolving the viral genotype determination in these samples. Our finding, together with other studies,^22,31^ proved that Xpert HCV VL could identify all major HCV genotypes in different parts of the world. As highly potent pangenotypic regimens for HCV treatment are not yet available in most countries, this assay may provide an important contribution for simplified diagnosis strategies to possibly skip the determination of the viral genotype in the cascade of care of HCV infection.

One of the study’s strengths was that it used samples from numerous locations across Indonesia, covering the most common HCV genotypes in Indonesia and its surrounding countries. In parallel, the study was used as a training forum of district laboratory technicians, who were directly supervised by research scientists at the Eijkman Institute in Jakarta. Thus, it exemplified a capacity-building project for decentralized service for HCV viral load determination.

Several other POC tests for HCV have been developed, including Xpert^®^ HCV Viral Load Finger-Stick (Xpert HCV VL FS), which can detect and quantify HCV RNA with high sensitivity and specificity from 100 μL of capillary whole blood in less than 60 minutes.^15,32,43^ However, this assay has not been approved by the FDA; and according to a preliminary study, further improvement and evaluation studies are still needed before it is ready for routine use.^44^ Another device is the Genedrive^®^ HCV (Genedrive Diagnostics, Manchester, United Kingdom), which can detect and semiquantify HCV RNA from 30 μL of plasma in less than 90 minutes.^45^ Despite WHO prequalification in May 2020, this test still requires a venous puncture for the collection of plasma samples and needs centrifugation, which is not easily accessible in remote areas.^46^ As a consequence of COVID-19, enormous public funding and health resources have been reallocated to respond to the pandemic around the world, disrupting the provision of essential services and evaluation studies of some tests in the communities.^15^ Therefore, in the current situation, the Xpert HCV VL test could be an option to support the “one-step diagnosis strategy”; testing HCV viremia can be performed at or near patient care by nonlaboratory-trained individuals such as physicians, nurses, and nursing assistants.^18,47^ A modeling study on the use of the Xpert system for screening and diagnostics in LMICs resulted in an increased diagnosis rate, with reduced overall cost of HCV elimination by 21%, in which the costly upfront initial investment of the system may lead to cost savings in the future.^48^ In Indonesia, the initial investment had happened through installation and localization of Xpert devices in several provinces as part of the TB,^28^ HCV,^27,28^ and current COVID-19 programs.^49,50^ The operational cost of the Xpert system is minimal because each sample can be processed individually without the need of batch testing,^19^ which would be more cost-effective for testing in remote settings.^51^ Further, the same-day results may improve linkage to care and prevent patient loss to follow-up.

We acknowledge several limitations in this study. The sample size could be a potential limitation. However, the sensitivity and specificity of Xpert HCV VL against the comparator assay was good and the correlation was strong, both among overall samples and by genotype. Further, a selection bias in participants recruited (i.e., patients with anti-HCV referred for treatment decision), who were more likely to be HCV RNA positive, could be one limitation. This also occurred in other studies, that most subjects were patients engaged in health services that could lead to a greater sensitivity and specificity of the test.^22,31,43^ Indeed, further studies on specimens from the general population had been planned in early 2020. However, this plan could not be realized because of the reallocation of resources and mobilization of health workforce to respond to the COVID-19 pandemic. Another limitation is that we did not include samples during or at the end of treatment that could represent those with low levels of quantifiable HCV RNA. Even though our finding showed that both platforms had comparable sensitivity, there were only a small number of samples in the low-range values. Additional study on samples during or post treatment would be useful to ensure the performance of Xpert HCV VL among low viral load samples.^22,31,52^

In conclusion, this study demonstrates a good performance of the Xpert HCV VL test as a POC platform comparable to that of a market-leading assay for a treatment decision and determining the outcome of HCV antiviral treatment. The robustness of the data coming from this study suggests that this assay can be used for decentralized HCV viral load testing, which would streamline the cascade of care for patients in areas with resource-limited settings. It is expected that the mobilization of COVID-19 investments, including the distribution of additional GeneXpert modular systems in LMICs at the time of writing this report, could ensure some continuity of HCV testing services, once the pandemic is controlled.^15,49,50^
